# CAMONCO 2: Results From a Randomized Controlled Trial Comparing Open Dialogue About Complementary Alternative Medicine and Standard Care on Patients' Quality of Life

**DOI:** 10.1200/OP.24.00345

**Published:** 2024-12-19

**Authors:** Mette Stie, Signe Timm, Charlotte Delmar, Birgitte Nørgaard, Lars Henrik Jensen

**Affiliations:** ^1^ Department of Oncology, University Hospital of Southern Denmark, Vejle, Denmark; ^2^ Department of Regional Health Research, Faculty of Health Sciences, University of Southern Denmark, Odense, Denmark; ^3^ Department of Public Health, Research Unit for Nursing and Health Care, Aarhus University, Aarhus, Denmark; ^4^ Department of Public Health, University of Southern Denmark, Odense, Denmark

## Abstract

The rising prevalence of complementary alternative medicine (CAM), including for example, dietary supplements, massage, and acupuncture, among patients with cancer underscores the necessity of integrating open dialogue about complementary alternative medicine (OD-CAM) into oncology treatment and care.

**PURPOSE:**

This randomized controlled trial aimed to investigate the impact of OD-CAM on patients' quality of life (QoL), emotional well-being, decision regret, and survival. Patients undergoing antineoplastic treatment were randomly allocated to receive standard care (SC) plus OD-CAM or SC alone. The primary end point was patient-reported QoL 8 weeks after enrollment. Secondary end points included patient-reported QoL, anxiety, depression, and decision regret at 12 and 24 weeks after enrollment and overall survival at 52 weeks after enrollment.

**MATERIALS AND METHODS:**

Patient-reported outcomes were evaluated using the European Organisation for Research and Treatment of Cancer Computer Adaptive Test Core questionnaire, the Hospital Anxiety and Depression questionnaire, and the Decision Regret Scale.

**RESULTS:**

A total of 210 patients were equally randomly assigned, leaving 105 patients in each group. No significant differences were observed in QoL, well-being, decision regret, or survival between the groups.

**CONCLUSION:**

OD-CAM did not demonstrate superiority over SC in enhancing the QoL and well-being of patients undergoing antineoplastic treatment. Increased levels of anxiety and fatigue might result from OD-CAM, underscoring the importance of the person-oriented approach inherent in OD-CAM. OD-CAM may hold clinical significance, especially for those already inclined toward CAM; however, further investigation into the essential components of OD-CAM and the characteristics of patients who are most likely to experience significant improvements of OD-CAM is recommended.

## INTRODUCTION

Worldwide, the utilization of complementary alternative medicine (CAM) among patients with cancer is widespread.^1^ Although lacking an official definition,^2^ CAM generally encompasses nonmainstream or alternative modalities such as specialized diets, herbal remedies, vitamin supplementation, mindfulness practices, acupuncture, yoga, massage, and spiritual healing, used alongside conventional medical treatments.^3^ A recent comprehensive systematic review incorporating 61 studies and representing over 20,000 patients with cancer reported a mean CAM usage at 51%,^1^ which is higher than what was reported in an earlier systematic review of studies conducted in 18 countries, suggesting that CAM use has increased considerably over the past years.^4^

**CONTEXT**

**Key Objective**
Does open dialogue about complementary alternative medicine (OD-CAM) integrated early in the oncology treatment and care benefit patients?
**Knowledge Generated**
In this study involving 210 patients with cancer, participating in a single session of OD-CAM with a nurse-specialist did not improve quality of life and well-being compared with patients receiving standard care.
**Relevance**
The study has shown that OD-CAM is feasible to integrate in daily oncology practice and may benefit patients already using complementary alternative medicine.

CAM is primarily used to complement conventional oncology treatment, enhancing well-being and supporting main treatment, rather than replacing standard care (SC).^1^ Its usage often continues after diagnosis,^5^ with chemotherapy and radiotherapy further increasing CAM adoption.^5-9^

The use of CAM presents both advantages and challenges. Some dietary supplements, like garlic and vitamins, can interfere with cancer treatments, potentially making them less effective.^10-13^ CAM therapies like acupuncture and mind-body techniques can improve quality of life (QoL) by reducing symptoms like nausea, pain, and fatigue (FA).^14-19^ Overall, CAM may enhance well-being and alleviate certain side effects of cancer treatment.^20^ Nevertheless, the level of evidence regarding CAM's efficacy varies, posing challenges for patients in discerning its potential benefits or risks. According to a systematic review encompassing 35 studies, the decision-making process regarding CAM is complex and nonlinear, influenced by patients' personal motivations, circumstances, diseases, and treatment stages.^21^ Consequently, an open and person-oriented dialogue incorporating evidence-based guidance on CAM should be an integral component of cancer treatment and care.^22-24^ Open dialogue about complementary alternative medicine (OD-CAM) in oncology has been shown to improve patient well-being and satisfaction.^25-29^ A previous randomized controlled trial suggested that early integration of OD-CAM in oncology treatment and care could reduce psychological stress and improve QoL and survival.^30^ However, evidence supporting its efficacy is limited, and its integration into cancer care is constrained. This randomized controlled trial aimed to examine the impact of OD-CAM on patients' individual QoL, emotional well-being, decision regret, and survival. It was hypothesized that OD-CAM would enhance these outcomes.

## MATERIALS AND METHODS

The study is reported according to the CONSORT guidelines.^31^

### Design

This is a nonblinded, parallel group, randomized controlled superior trial evaluating the efficacy of OD-CAM compared with SC in enhancing the QoL of patients receiving oncology treatment and care. The study was registered prospectively with ClinicalTrials.gov (identifier: NCT04299451) and approved by the Danish Data Protection Agency under the Region of Southern Denmark (20/11100). The procedures conducted in this study adhere to the principles outlined in the Declaration of Helsinki.^32^ No changes to methods or interim analysis were made during the study. The materials and methods are described in detail elsewhere.^33^

### Participants

Patients age 18 years or older, diagnosed with primary cancer or recurrence within the last 3 months, and planned antineoplastic treatment for at least 2 months were eligible for inclusion. Since CAM is used by patients with various types of cancer^1^ and all may benefit from dialogue about CAM, the participants were not limited to a specific diagnosis. Additionally, individuals were required to have an expected life expectancy of at least 6 months, be proficient in Danish (spoken and written), and provide signed informed consent. Eligible patients were informed about the study and invited to participate before their second cycle of oncology treatment. Signed consent had to be obtained within 12 weeks from the start of treatment and before random assignment.

### Setting

The study was conducted at the Outpatient Oncology Department of Vejle Hospital, Lillebaelt Hospital, Denmark, from May 2020 to January 2023. This department delivers treatment and care to patients with breast, gynecological, prostate, pulmonary, colorectal, anal, and pancreatic cancers. Notably, the department does not currently offer any CAMs.

### Intervention Group (OD-CAM)

Patients in the intervention group received SC and participated in one session of OD-CAM. The sessions were led by a nurse-specialist with expertise in integrative medicine, having completed the Fellowship in Integrative Medicine program at the University of Arizona. In the OD-CAM sessions, the nurse-specialist integrates patients' values, preferences, reasoning, will, concerns, beliefs, feelings, needs, and lifestyles with evidence-based information about CAM. This guidance aims to ensure the safe and healthy utilization of CAM by patients. Further details of the intervention are described in Table 1 and previous studies.^30,33^

**TABLE 1.** Guideline for OD-CAM

Setting for the OD-CAM	
Preparation	The patient is encouraged to prepare for the session, including considerations as to current and future use of CAM
Environment	The OD-CAM takes place away from the clinic in a consultation room designed specifically to provide a healing environment with soft and natural lighting, flowers, and relaxing furniture
Schedule	The OD-CAM must be conducted no later than 2 weeks after random assignment and is scheduled to last 45-60 minutes
Nurse specialist	The nurse specialist has completed the program Fellowship in Integrative Medicine at the University of Arizona. This is a training program for health professionals in empowering individuals and communities to optimize health and well-being through evidence-based, sustainable, and integrative approaches
Integrative	This involves a healing-oriented approach viewing and respecting patients as whole and unique physical, emotional, social, and spiritual beings with values, knowledge, preferences, and beliefs. It aims to optimize health, QoL, and clinical outcomes and support patients to become active participants in their own healing and health. It emphasizes the therapeutic relationship between the health professional and the patient. On the basis of evidence, CAM information is provided alongside conventional cancer treatment

Content of the OD-CAM	In Cooperation With the Patient	Examples of Questions to Ask
Understand	Elicit the patients' understanding of their situation Clarify information preferences before asking about CAM use Ask open questions focusing on psychological/existential issues	What is your understanding of the situation at this point? What concerns you most about your illness and treatment? What are your hopes for the future?
Respect	Respect cultural, linguistic, and belief diversity Awareness of attitudes and information needs in relation to models of illness and treatment	What do you believe might have caused your illness?
Ask	Ask questions about CAM use Adopt an inquisitive, open-minded, and nonjudgmental approach Clarify reasons for asking about CAM	Are you currently doing or considering doing anything else for your condition/side effects, your overall health, or well-being? Are you taking any other medications or treatments? It is very important for me to know about any initiatives you have taken to address your illness so I can help you the best way possible. I am not an expert in this (CAM), but it is important to make sure that any actions or medications you take do not interact negatively with the treatment we give you
Explore (if the patient is already/considering using CAM)	Explore the details of CAM use and listen actively Enquire about current and planned CAM use Ask about reasons for and expected outcomes of CAM use Ask about expected outcomes of conventional treatment Ask if there is a provider of the CAM (if relevant), who it is, and what their role will be in relation to the CAM use Explore the evidence for the CAM's efficacy and safety Provide balanced evidence advice in relation to the CAM. Help respond to advice from family and friends (if relevant)	Can you tell me more about this CAM, please? What does it involve? How often do you use it? Have you used it before? What are your reasons for using this CAM? Which results do you hope for from this CAM? Has it been helpful so far? How do you know it is helpful for you? Who are you seeing for this CAM? (If relevant) Do you know if there has been any research on the effect of this CAM? Others want the best for you. Let's talk about these suggestions. What do you think of these suggestions?
Respond	Respond to the patient's emotional state, encourage expression of feelings Express empathy Support the desire for hope and control; address issues the patient seeks to influence by using CAM (eg, symptom control, alleviation of side effects, control, desire to live longer)	How are you feeling emotionally? How are you coping with your situation? It sounds like you want to do everything possible. It is natural to feel a need to explore the possible options and I fully support you in that (if relevant)
Discuss	Discuss relevant concerns about CAM while respecting the patient's beliefs Possible concerns Caution about substances with unknown effect and quality, high financial or time cost for CAM of unknown benefits, potential for psychological harm Discuss a reasonable trial period over which an assessment can be made regarding benefits/efficacy of CAM A symptom diary may help determine whether the CAM is beneficial for the individual patient Explore alternative ways of addressing the patient's underlying needs, hopes, or fears (especially if there are concerns about the CAM's potential harms)	I believe there is little evidence on the benefit or harm associated with this CAM. So, we should be cautious Might the time involved prevent you from doing other things you like to do? How do you think you might feel if you followed this advice (CAM use) but did not achieve the outcome you hoped for? How long would you expect it to take to see a benefit from this CAM? I can see that you hope this CAM will help you/your cancer/symptoms/side effects/well-being There are other options we can look at, too. Would you like to hear about them?
Advise	Encourage use of a CAM that may be beneficial Accept use of CAM for which there is no evidence of physical harm or benefit. Support the decision, although it conflicts with your private view Discourage use of CAM where there is no good evidence. It will be unsafe or harmful Particularly, discourage use of unproven CAM if it is to be used in place of potentially beneficial treatment, especially potentially curative treatment Balance advice with an acknowledgment of the patient's rights for self-determination and autonomy	I recommend this CAM, the evidence suggests that it could help you We do not know much about this CAM, but it does not seem to be harmful and it may even help you. I respect that is what you wish to do I have to be honest with you. I am concerned that this CAM may do you greater harm than good I respect and support your right to make this decision. However, I firmly believe that you have a better chance of a good outcome if you follow this treatment plan. Although there is little evidence for us to know if this CAM will be helpful, of course the decision is yours
Summarize	Summarize main points of discussion and check patient's understanding Provide websites and other information or resources, eg, information about dietary supplements, breathing exercises, yoga, meditation	We have covered a lot today. Just so that I can check that I have explained things properly, can you summarize what we have discussed? Do you have any further questions or issues you would like to discuss?
Document	Document the discussion in the patient's medical record and send a copy to the patient	I will document what we have discussed today in your medical record, and you will receive a copy in your e-Boks
Follow-up	Follow-up discussion about CAM, if relevant	

Abbreviations: CAM, complementary alternative medicine; OD-CAM, open dialogue about complementary alternative medicine; QoL, quality of life.

### Control Group (SC)

The control group received SC which involved antineoplastic treatment and supportive care at the oncology department. SC also encompassed ongoing assessment and monitoring of performance status, side effects, and symptoms. Additionally, patients were provided with information about a temporary website in Danish, which was part of SC, offering details on the effects of CAM.^34,35^

### Outcome Measures and Data Collection

The primary outcome measure was patient-reported QoL 8 weeks after enrollment with secondary measures at 12 and 24 weeks, alongside survival analysis.

QoL was measured using the European Organisation for Research and Treatment of Cancer Computer Adaptive Test Core questionnaire (EORTC QLQ CAT Core), which offers tailored assessments across 15 domains using pools of 262 validated questions.^36,37^

On the basis of the results from our previous study,^30^ QoL was predefined to encompass aspects of physical functioning (PF), emotional functioning (EF), fatigue (FA), and nausea and vomiting (NV). PF and EF are functional scales, whereas FA and NV are symptom scales.^38-41^

Emotional well-being was evaluated using the Hospital Anxiety and Depressions Questionnaire,^42^ and decision regret concerning conventional treatment was assessed using the Decision Regret Scale.^43^

Survival was determined for each patient 52 weeks after their inclusion in the study.

### Sample Size

In this study, an average difference of 10 points on the QoL scale between the intervention group and the control group 8 weeks after enrollment was deemed clinically significant, forming the basis for sample size calculation. With an estimated standard deviation (SD) of 24.2 and assuming a normal distribution of responses within each group, standard recommendations for randomized trials were followed. A type I error rate of 0.05 and a power of 0.80 were used. To detect a difference of 10 points and reject the null hypothesis of equal means between the two groups, inclusion of 93 subjects in each group (experimental and control) was required. Accounting for an expected dropout rate of 10%, the total number of patients to be included was 207.

### Random Assignment

Patients were randomly assigned in a 1:1 ratio to receive either SC plus the intervention (OD-CAM) or SC alone using the Research Electronic Data Capture (REDCap) system hosted by the OPEN organization. Allocation concealment was maintained through the OPEN Randomize service, which withheld the randomization code until all baseline data had been uploaded into REDCap. Patients assigned to the OD-CAM group were provided with a letter detailing the date, time, and location of their OD-CAM session, along with instructions on how to prepare for it. Patients randomly assigned to the SC group received a pamphlet directing them to a temporary website in Danish providing information about the effects of CAM.^34,35^ Oversight of REDCap procedures and allocation information was managed by a research nurse.

### Statistics

Patient characteristics were described using counts (n) and proportions (%) for categorical variables, and means with SD for numerical variables. Normality of data distribution was assessed using histogram plots. QoL and well-being were presented as mean scores with 95% CI, and comparisons between the two groups were conducted using *T* test.

As the data on the NV domain of the EORTC QLQ CAT Core score did not follow a normal distribution, the result was reported as a median, and comparisons were made using the Wilcoxon rank-sum test. Similarly, the decision regret score was also reported as a median, and comparisons were conducted using the Wilcoxon rank-sum test. To investigate whether certain subgroups of patients benefited more, less, or not at all from the OD-CAM intervention, chi-square tests were stratified by patients receiving curative treatment versus palliative treatment, as well as users versus nonusers of CAM. Selection of subgroups were based on previous research.^1^

Overall survival was assessed using a log-rank test 52 weeks after inclusion for each patient. It was assumed, based on the study by Lyhne et al,^44^ that missing data were missing at random, and therefore, all analyses were performed on complete cases.

### IRB Statement

According to the Committee on Health Research Ethics for Southern Denmark, ethics approval of this study is not required (S-20202000-5, 20/1019). The Region of Southern Denmark (Journal no. 20/11100) approved the storing and handling of data.

## RESULTS

**FIG 1.**
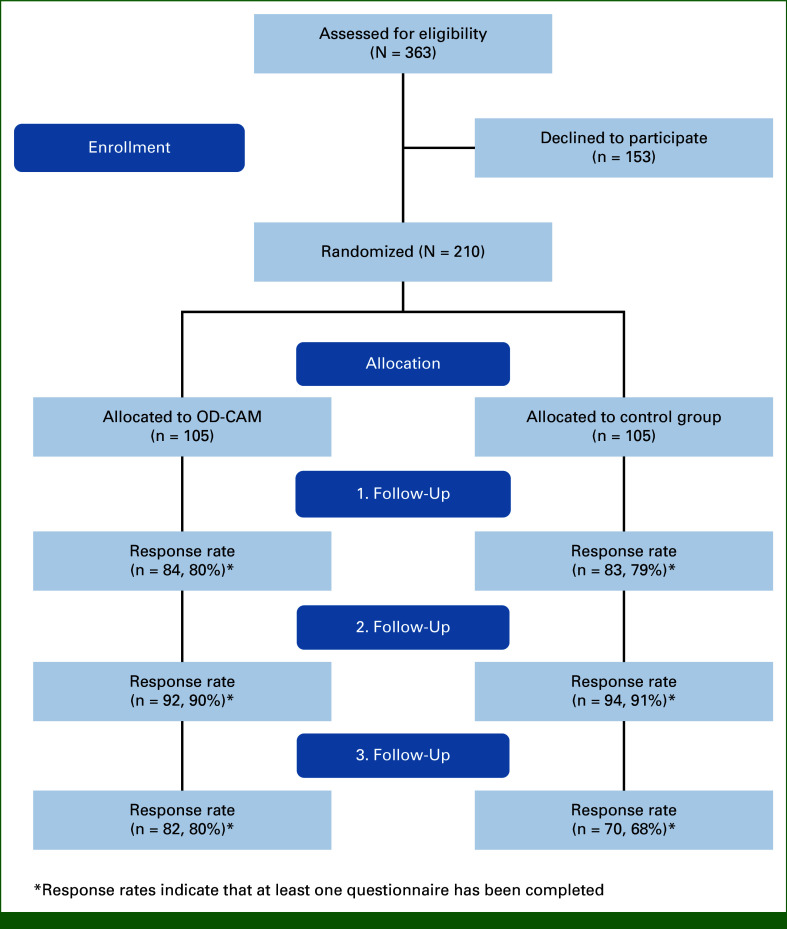
Flow diagram. ^a^Response rates indicate that at least one questionnaire has been completed. OD-CAM, open dialogue about complementary alternative medicine.

Of the 363 eligible patients, 210 patients were included and randomly assigned to receive either OD-CAM (n = 105) or SC (n =105; Fig 1). Demographic data, clinical characteristics, and attitudes toward and use of CAM were comparable between the two groups (Table 2). All patients assigned to the intervention group participated in one session of OD-CAM.

**TABLE 2.** Patient Characteristics

Characteristics	Intervention Group (n = 105)	Control Group (n = 105)	All (N = 210)
Sex, No (%)			
Female	62 (59)	46 (53)	118 (56)
Male	43 (41)	49 (47)	92 (44)
Age, mean (SD)	60.9 (11.8)	60.7 (12)	60.8 (11.9)
Married/in a relationship, No (%)			
Yes	79 (75)	81 (77)	160 (76)
No	19 (18)	19 (18)	38 (18)
Other	3 (3)	1 (1)	4 (2)
Missing	4 (4)	4 (4)	8 (4)
Education, No (%)			
High	7 (7)	11 (10)	18 (9)
Middle	72 (69)	71 (68)	143 (68)
Low	16 (15)	14 (13)	30 (14)
Other	4 (4)	5 (5)	9 (4)
Missing	6 (5)	4 (4)	10 (5)
Cancer diagnosis, No (%)			
Breast	29 (27)	27 (26)	56 (26)
Prostate	17 (16)	12 (10)	29 (14)
Lung	21 (20)	26 (25)	47 (22)
GI	26 (25)	25 (24)	51 (24)
Ovarian	4 (4)	4 (4)	8 (4)
Uterine	0	1 (1)	1 (1)
Pancreas	4 (4)	6 (6)	10 (5)
Missing	4 (4)	4 (4)	8 (4)
Treatment intention, No (%)			
Curative	50 (48)	38 (36)	88 (42)
Palliative	50 (48)	63 (60)	113 (54)
Missing	5 (4)	4 (4)	9 (4)
Current treatment, No (%)			
Chemotherapy	75 (70)	68 (64)	143 (67)
Immunotherapy	6 (6)	7 (7)	13 (6)
Antibody therapy	2 (2)	1 (1)	3 (1)
Chemotherapy + antibody	7 (7)	12 (11)	19 (9)
Chemotherapy + radiation	7 (7)	6 (6)	13 (6)
Other	4 (4)	7 (7)	11 (5)
Missing	4 (4)	4 (4)	8 (4)
Attitude toward CAM, No (%)			
Don't know	5 (5)	8 (8)	13 (6)
Against	1 (1)	1 (1)	2 (1)
Neutral	20 (19)	18 (17)	38 (18)
Positive	41 (39)	41 (39)	82 (39)
Very positive	34 (32)	33 (31)	67 (32)
Missing	4 (4)	4 (4)	8 (4)
Use of CAM, No (%)			
Yes	73 (70)	78 (74)	151 (72)
No	27 (26)	23 (22)	50 (24)
Don't know	1 (1)	0	1 (0)
Missing	5 (3)	4 (4)	9 (4)
CAM type, No (%)			
Nutrition^a^			
None	45 (42)	36 (34)	81 (38)
One type	52 (50)	61 (58)	113 (54)
Two types	8 (8)	8 (8)	16 (8)
Psychological/physical approaches, No (%)^b^			
None	67 (63)	74 (70)	141 (67)
One type	25 (24)	13 (12)	38 (18)
Two types	10 (10)	12 (11)	22 (10)
Three types	2 (2)	5 (5)	7 (4)
Four types	1 (1)	1 (2)	2 (1)
Other, No (%)^c^			
None	88 (83)	83 (79)	171 (81)
One type	17 (17)	19 (18)	36 (17)
Two types	0	3 (3)	3 (2)

Abbreviations: CAM, complementary alternative medicine; SD, standard deviation.

^a^ Nutrition: special diets, herbs, vitamins, minerals, dietary supplements, and probiotics.

^b^ Psychological/physical approaches: mindfulness, tai chi, yoga, acupuncture, massage therapy, spinal manipulation, art therapy, music therapy, dance, chiropractic and osteopathic manipulation, meditation, relaxation techniques, qigong, hypnotherapy, and pilates.

^c^ Other: traditional healing, ayurvedic medicine, traditional Chinese medicine, homeopathy, naturopathy, and functional medicine.

The response rates remained high throughout the study period, with 80% and 79% at the first follow-up (8 weeks), 90% and 91% at the second follow-up (12 weeks), and 80% and 68% at the third follow-up (24 weeks) for the OD-CAM group and SC group, respectively (Fig 1).

The primary end point, QoL score at the first (8 weeks) follow-up, was 53.20 (95% CI, 50.78 to 55.63) in the OD-CAM group and 53.64 (95% CI, 51.33 to 55.95) in the SC group (*P* = .79). QoL assessments beyond the first evaluation showed no significant disparities between the two groups over the study duration.

Key results regarding secondary end points reveal no significant differences in EF, PF, NV, FA, anxiety, depression, and total anxiety and depression scores between the intervention and control groups across all evaluated time frames.

During the second (12 weeks) and third (24 weeks) follow-up, EF domain score was, respectively, 51.07 (95% CI, 49.12 to 53.03) and 50.21 (95% CI, 48.14 to 52.28) in the OD-CAM group and 49.36 (95% CI, 47.24 to 51.47) and 49.52 (95% CI, 47.14 to 51.91) in the SC group. Similar results were identified in the PF domain score, which was 47.46 (95% CI, 45.45 to 49.46) and 48.05 (95% CI, 46.21 to 59.89) in the OD-CAM group and 46.04 (95% CI, 43.74 to 48.34) and 46.27 (95% CI, 43.91 to 48.63) in the SC group at second and third follow-up, respectively. Differences were not statistically significant at any follow-up.

Total anxiety and depression scores were, respectively, 8.00 (95% CI, 6.53 to 9.47), 8.20 (95% CI, 6.64 to 9.77), and 8.39 (95% CI, 6.77 to 10.00) in the OD-CAM group and 7.58 (95% CI, 6.31 to 8.85), 7.5 (95% CI, 5.94 to 9.06), and 7.29 (95% CI, 5.74 to 8.85) in the SC group during the follow-up period. However, no significant differences were found between the groups at any follow-up or at any of the anxiety and depression subscales. The anxiety score was notably higher compared with depression in both groups (Table 3).

**TABLE 3.** QoL, Emotional Well-Being, and Decision Regret

Outcome Measures/Follow-up	Intervention Group, 95% CI	Control Group, 95% CI	*P*
Overall QoL			
8 weeks (n = 81/79)	53.20 (50.78 to 55.63)	53.64 (51.33 to 55.95)	.79
12 weeks (n = 82/78)	54.04 (51.84 to 56.25)	53.04 (50.24 to 55.85)	.58
24 weeks (n = 80/68)	51.81 (49.56 to 54.07)	51.41 (48.32 to 54.51)	.83
Emotional functioning			
8 weeks (n = 81/79)	48.91 (47.01 to 50.80)	49.59 (47.83 to 51.36)	.59
12 weeks (n = 82/78)	51.07 (49.12 to 53.03)	49.36 (47.24 to 51.47)	.24
24 weeks (n = 80/68)	50.21 (48.14 to 52.28)	49.52 (47.14 to 51.91)	.66
Physical functioning			
8 weeks (n = 81/79)	47.05 (45.14 to 48.93)	47.67 (45.84 to 49.48)	.64
12 weeks (n = 82/78)	47.46 (45.45 to 49.46)	46.04 (43.74 to 48.34)	.36
24 weeks (n = 80/68)	48.05 (46.21 to 59.89)	46.27 (43.91 to 48.63)	.50
Nausea/vomiting^a^			
8 weeks (n = 81/79)	51.74 (51.74-53.90)	51.74 (51.74-51.74)	.53^b^
12 weeks (n = 82/78)	51.74 (51.74-51.74)	51.74 (51.74-51.74)	.58^b^
24 weeks (n = 80/68)	51.74 (51.74-51.74)	51.74 (51.74-56.96)	.55^b^
Fatigue			
8 weeks (n = 81/79)	56.15 (54.56 to 57.75)	55.79 (54.11 to 57.48)	.76
12 weeks (n = 82/78)	55.49 (53.63 to 57.34)	55.38 (53.35 to 57.40)	.94
24 weeks (n = 80/68)	55.12 (53.05 to 57.19)	54.22 (51.87 to 56.57)	.56
Anxiety			
8 weeks (n = 80/66)	4.92 (4.03 to 5.82)	4.80 (3.96 to 5.64)	.81
12 weeks (n = 79/69)	4.72 (3.80 to 5.64)	4.58 (3.60 to 5.56)	.83
24 weeks (n = 75/62)	5.09 (4.11 to 6.08)	4.89 (3.86 to 5.91)	.77
Depression			
8 weeks (n = 80/67)	3.08 (2.38 to 3.77)	2.73 (2.13 to 3.33)	.46
12 weeks (n = 79/69)	3.48 (2.68 to 4.28)	2.88 (2.21 to 3.56)	.26
24 weeks (n = 75/61)	3.30 (2.55 to 4.04)	2.49 (1.84 to 3.14)	.12
Total anxiety and depression			
8 weeks (n = 80/66)	8.00 (6.53 to 9.47)	7.58 (6.31 to 8.85)	.67
12 weeks (n = 79/69)	8.20 (6.64 to 9.77)	7.5 (5.94 to 9.06)	.53
24 weeks (n = 75/61)	8.39 (6.77 to 10.00)	7.29 (5.74 to 8.85)	.34
Decision regret^a^			
Baseline (n = 98/96)	0 (0-15)	0 (0-20)	.47^b^
8 weeks (n = 80/65)	5 (0-20)	5 (0-20)	.99^b^

Abbreviation: QoL, quality of life.

^a^ Median and percentiles (25th percentile-75th percentile).

^b^ Wilcoxon rank-sum test.

Moreover, the study explored decision regret at baseline and 8 weeks, yielding median scores that further emphasize the lack of significant distinction in this domain as well, with *P* values of .47 and .99, respectively. The median score for decision regret was 5 (25th percentile-75th percentile: 0-20) in both groups at the first follow-up (Table 3).

Regarding survival, only one patient in the intervention group and two patients in the control group died within 1 year after inclusion.

Results from the predefined subgroups, categorized by curative versus palliative treatment and CAM usage versus nonusage, are presented in Table 4. At the first follow-up (8 weeks), the EF domain was significantly (*P* = .02) higher among nonusers of CAM in the SC group (52.33 [95% CI, 48.22 to 56.44]) compared with nonusers in the OD-CAM group (46.42 [95% CI, 43.31 to 49.53]). Similarly, CAM nonusers receiving SC reported a significantly (*P* = .04) higher level of overall QoL (56.79 [95% CI, 51.99 to 61.60]) compared with nonusers in the OD-CAM group (49.84 [95% CI, 44.95 to 54.73]). There was no significant difference observed between subgroups of patients receiving curative or palliative treatment.

**TABLE 4.** Subgroup Analysis

Follow-Up			
Exposure Groups	Intervention (OD-CAM), 95% CI	Control (SC), 95% CI	*P*
Curative treatment (n = 37/29)			
Overall			
QoL	52.55 (49.02 to 56.08)	53.89 (50.06 to 57.72)	.60
EF	49.14 (46.72 to 51.57)	49.29 (46.46 to 52.13)	.93
PF	50.60 (47.70 to 53.50)	50.33 (47.32 to 53.34)	.89
NV^a^	51.74 (51.74-57.51)	51.74 (51.74-51.74)	.68^b^
FA	56.83 (54.78 to 58.87)	55.67 (52.57 to 58.77)	.51
Palliative treatment (n = 47/41)			
Overall			
QoL	54.30 (50.66 to 57.95)	53.20 (50.07 to 56.35)	.65
EF	49.41 (47.34 to 52.07)	49.70 (47.33 to 52.07)	.88
PF	44.25 (41.98 to 46.52)	45.76 (43.52 to 48.01)	.34
NV^a^	51.74 (51.74-51.74)	51.74 (51.74-51.74)	.65^b^
FA	55.31 (52.71 to 57.91)	55.76 (53.58 to 57.94)	.78
CAM nonusers (n = 20/17)			
Overall			
QoL	49.84 (44.95 to 54.73)	56.79 (51.99 to 61.60)	.04
EF	46.42 (43.31 to 49.53)	52.33 (48.22 to 56.44)	.02
PF	45.45 (41.36 to 49.55)	47.64 (42.26 to 53.02)	.49
NV^a^	51.74 (51.74-64.56)	51.74 (51.74-51.74)	.39^b^
FA	57.90 (55.21 to 60.60)	55.68 (52.26 to 59.10)	.28
CAM users (n = 59/59)			
Overall			
QoL	53.69 (50.93 to 56.46)	53.18 (50.66 to 55.69)	.78
EF	49.38 (47.05 to 51.71)	48.87 (46.85 to 50.88)	.74
PF	47.28 (45.13 to 49.43)	47.85 (46.02 to 49.67)	.69
NV^a^	51.74 (51.74-53.90)	51.74 (51.74-57.12)	.83^b^
FA	56.04 (54.14 to 57.94)	55.96 (53.95 to 57.97)	.96

Abbreviations: CAM, complementary alternative medicine; EF, emotional functioning; FA, fatigue; NV, nausea and vomiting; OD-CAM, open dialogue about complementary alternative medicine; PF, physical functioning; QoL, quality of life; SC, standard care.

^a^ Median and percentiles (25th percentile - 75th percentile).

^b^ Wilcoxon rank-sum test.

## DISCUSSION

This prospective randomized controlled trial is the first comprehensive study to investigate the impact of OD-CAM on the QoL of patients with cancer using the EORTC QLQ CAT Core. The majority of patients with cancer use CAM, and addressing this in oncology is challenging. Insights from this study may help update oncology practices to provide equitable, high-quality, value-based care.

On the basis of the previous studies,^26,29,30^ we hypothesized that OD-CAM could improve patients’ QoL. By comparing patients participating in one session on OD-CAM along with SC with those receiving SC alone, we found that QoL was similar between the groups.

Other research works suggest that OD-CAM might improve QoL and well-being over the long term.^27,45^ Patients often turn to CAM to improve health and well-being,^1,46,47^ and OD-CAM aims to empower patients in this regard. By facilitating discussions on safe and healthy CAM practices, OD-CAM may indirectly contribute to better overall QoL in the long term. The results of our study did, however, not support these findings.

Although OD-CAM was anticipated to affect survival outcomes, the study's limited number of deaths within 1 year (only one patient in the OD-CAM group and two patients in the SC group) prevented significant associations from being identified.

The lack of difference on anxiety and depression between the two groups contrasts with previous studies suggesting that counseling on CAM may address patients' concerns^25^ and alleviate psychological distress.^27^ Additionally, an observational study found that discussions about CAM contain more psychological statements compared with dialogues where CAM is not discussed,^48^ indicating that OD-CAM potentially alleviates psychological concerns.

Although not significant, we observed a higher level of anxiety compared with depression in both groups, and similar to studies on communication patterns between oncologists and patients, higher anxiety levels were observed within CAM discussions.^49^ This may be due to the association between CAM use, fear, and uncertainty.^50^ The high prevalence of CAM use in both groups (70% and 74%) might contribute to increased anxiety, but does not fully explain the higher anxiety level in the OD-CAM group compared with the SC group.

Strongly emphasizing patient empowerment through CAM discussions could inadvertently burden patients. Although it is unclear whether this occurred in our study, it aligns with research cautioning against overemphasizing patient agency in a self-care–focused health care system.^51-53^ Although anxiety and depression were not significantly higher in the OD-CAM group, this underscores the importance of a person-oriented approach in OD-CAM, where health professionals sensitively consider and respect patient's situation and resources to prevent psychological distress.

Studies of communication about CAM in cancer care highlight that open, respectful dialogue, where health professionals consider patients' values, is crucial for shared decision making and reduces decision regret.^54^ We anticipated that OD-CAM, focusing on patients’ values, preferences, reasoning, concerns, beliefs, feelings, needs, and lifestyles, would affect decision regret. However, like Chong et al,^55^ we found no impact, possibly due to data collection timing (8 weeks after enrollment).

A word of caution: subgroup analysis showed that nonusers of CAM in the SC group reported significantly higher EF and overall QoL compared with nonusers in the OD-CAM group. Despite the risk of type I error, these clinically relevant differences suggest that nonusers may not benefit from OD-CAM, whereas CAM users might. These findings should be considered when planning future studies on the impact of OD-CAM and integrating OD-CAM into oncology practice.

We explored whether patients receiving palliative treatment in the OD-CAM group would report higher QoL than those receiving curative treatment. Given higher CAM use among palliative patients,^56^ we expected OD-CAM to be more beneficial for them. However, our subgroup analysis found no difference between palliative and curative treatment groups.

This study, with its prospective randomized design, involving 210 patients and high intervention completion rates, represents the most comprehensive trial on integrated OD-CAM in oncology. The OD-CAM was consistently facilitated by the same nurse-specialist, ensuring intervention homogeneity. However, the study has important limitations.

Blinding was not feasible due to the interventional nature, potentially introducing bias. Notification on the website may have raised awareness about CAM, thereby minimizing the impact of the OD-CAM. Additionally, measuring QoL and well-being using predefined questionnaires may have captured indirect outcomes rather than proximal outcomes like relationship and trust, which can affect outcomes like self-care and QoL.^57^

Although the randomized controlled design provided insights, it did not fully explore essential elements of the OD-CAM such as the specific impact of the nurse-specialist's approach, engagement and communication style, or patients' expectations. Qualitative studies exploring patients' experiences could provide valuable insights to these aspects.

The intervention consisting a single OD-CAM session may be viewed as limited, yet practical for daily clinical practice. Conducted during the overwhelming treatment initiation phase, it may have restricted information uptake, potentially contributing to the lack of significant impact observed.

Furthermore, the results are specific to patients undergoing oncology treatment, without exploration of OD-CAM's effect on cancer survivors or those in the terminal phase. The study's heterogeneous cancer diagnosis, the predominance of CAM users with positive attitude toward CAM, and the lack of data on participants' race and ethnicity limit its generalizability. Research is needed to compare nonparticipants’ characteristics with those included, clarifying OD-CAM's specific benefits for CAM users and guiding its integration into future oncology treatment and care.

Finally, being conducted at a single center limits the broader applicability of the results to other settings.

In conclusion, in this study, OD-CAM did not demonstrate superiority over SC in improving patients' QoL, well-being, and survival. Furthermore, OD-CAM did not reduce patients' decision regret compared with SC, and it is important to note a risk of OD-CAM negatively affecting EF and QoL in patients who do not use CAM. The person-oriented approach of the health professional facilitating the OD-CAM is pivotal in ensuring its effectiveness and minimizing potential negative effects.

In oncology practice, OD-CAM is considered safe for patients who are already incorporating CAM into their oncology treatment and care. Future research should focus on identifying the critical elements of the OD-CAM and their potential impact on patients' QoL, well-being, and decision making about both conventional oncology treatment and CAM. Additionally, it is crucial to improve the identification of which patients benefit the most from OD-CAM.
